# A Microhomology-Mediated Break-Induced Replication Model for the Origin of Human Copy Number Variation

**DOI:** 10.1371/journal.pgen.1000327

**Published:** 2009-01-30

**Authors:** P. J. Hastings, Grzegorz Ira, James R. Lupski

**Affiliations:** 1 Department of Molecular and Human Genetics, Baylor College of Medicine, Houston, Texas, United States of America; 2 Department of Pediatrics, Baylor College of Medicine, Houston, Texas, United States of America; 3 Texas Children's Hospital, Houston, Texas, United States of America; Université Paris Descartes, INSERM U571, France

## Abstract

Chromosome structural changes with nonrecurrent endpoints associated with genomic disorders offer windows into the mechanism of origin of copy number variation (CNV). A recent report of nonrecurrent duplications associated with Pelizaeus-Merzbacher disease identified three distinctive characteristics. First, the majority of events can be seen to be complex, showing discontinuous duplications mixed with deletions, inverted duplications, and triplications. Second, junctions at endpoints show microhomology of 2–5 base pairs (bp). Third, endpoints occur near pre-existing low copy repeats (LCRs). Using these observations and evidence from DNA repair in other organisms, we derive a model of microhomology-mediated break-induced replication (MMBIR) for the origin of CNV and, ultimately, of LCRs. We propose that breakage of replication forks in stressed cells that are deficient in homologous recombination induces an aberrant repair process with features of break-induced replication (BIR). Under these circumstances, single-strand 3′ tails from broken replication forks will anneal with microhomology on any single-stranded DNA nearby, priming low-processivity polymerization with multiple template switches generating complex rearrangements, and eventual re-establishment of processive replication.

## Introduction

In the past few years, we have learnt that a major component of the differences between individuals is variation in the number of copies of segments of the genome, and of genes included in these segments (copy number variation or CNV) (for definition of abbreviations, see Table 1). A considerable portion of the genome is involved in CNV [1]–[11]—with estimates of up to 12% [4]—which can arise meiotically and also somatically as shown by the finding that identical twins can differ in CNV [12]. CNV has been a significant component of primate evolution [13]–[16]. Here we draw on evidence on the mechanism of DNA transactions in *Escherichia coli*, yeast, *Drosophila*, mammals, and human cancer to derive a model for the origin of CNV based on the mechanism of BIR occurring at sites of microhomology (microhomology-mediated BIR or MMBIR).

**Table 1:** Abbreviations Used in the Text.

Abbreviation	Meaning
BIR	Break-induced replication, a recombination-based mechanism for restarting broken replication forks.
CNV	Copy number variation, variation within a population of the number of copies of a gene or length of genome.
DSB	Double-strand break, a break in both strands of a DNA molecule.
FoSTeS	Fork stalling and template switching, a replicative mechanism for changing chromosome structure.
LCR	Low copy repeat, a length of genome that occurs twice or a few times.
MMBIR	Microhomology-mediated break-induced replication, a replication-based mechanism of recombination between sequences with very little base identity, proposed here.
NAHR	Nonallelic homologous recombination, homologous recombination occurring between low copy repeats.
NHEJ	Nonhomologous end joining, a mechanism for repair of DNA double-strand breaks that does not require homology.
SD	Segmental duplication, a repetition of a length of genome.

## Genomic Disorders

**Figure 1:**
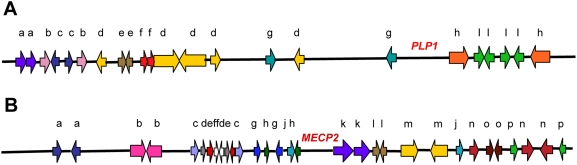
In silico analyses revealed complex genomic architecture in regions of nonrecurrent rearrangement. (A) The ∼3 Mb surrounding the *PLP1* gene and (B) the ∼4 Mb surrounding the *MECP2* gene on the X chromosome contain numerous LCRs in various orientations [33],[106]. LCRs are represented by the colored block arrows, and like LCR copies are designated by color and letter for a given sequence. Orientation is depicted by the direction of the block arrow.

Although we can see that considerable variation in copy number is tolerated or is advantageous to its carrier, some genes are dosage-sensitive, and duplication or deletion involving these genes gives rise to human clinical phenotypes collectively referred to as genomic disorders [17]. This has allowed the ascertainment of structural changes and thus the study of the origin of CNV. For recurrent rearrangements, much CNV stems from homologous recombination between segments that already occur as two or more copies. When this happens, sequences that lie between the repeats that recombine will be either duplicated or deleted, thus changing the copy number. This process is referred to as nonallelic homologous recombination, or NAHR [18]. The repeated sequences that recombine might occasionally be highly repetitive sequences that occur widely in the human genome [19] but are usually sequences that occur only twice or a few times (i.e., low-copy repeats, LCRs, or segmental duplications, SDs). The LCRs tend to occur in clusters in highly complex regions of the genome. These repeated segments might be short (about 10 kilobases (kb)), or up to several hundreds of kb in length, and they occur in either orientation. Some examples of genomic complex regions are shown in Figure 1.

The endpoints of CNVs that arose by NAHR occur in a few positions where there is sufficient homology for homologous recombination. Although many genomic disorders arise by NAHR [20], some rearrangements have endpoints in many different positions. These CNVs arose de novo by rearrangements at sites that lack extensive homology. Recent evidence on the distribution of nonpathological CNVs in two individuals suggests that most differences in copy number from the reference sequence arose by nonrecurrent events [2]. Thus nonrecurrent chromosomal changes arise quite frequently [21]. Because the nonrecurrent events presumably reflect the origin of most genome complexity, the study of them is important to the understanding of genomic disorders, genetic variability due to CNV, and human evolution.

**Figure 2:**
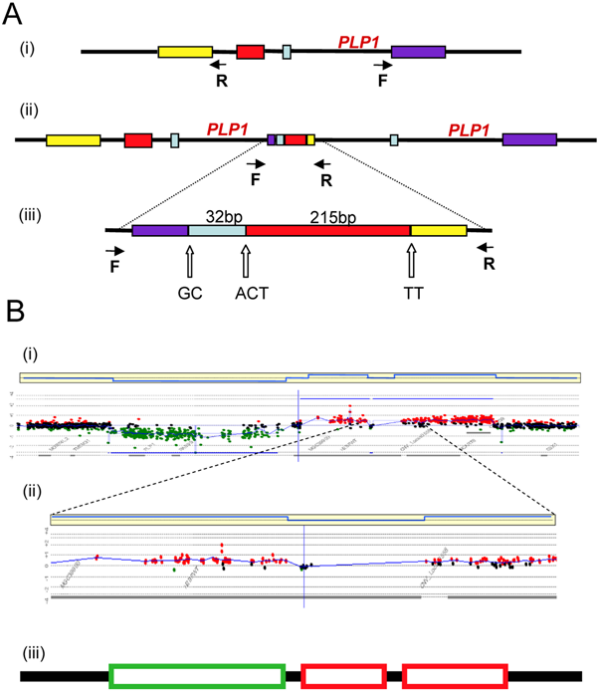
Complex rearrangements involving *PLP1* detected by junction analysis (A) and oligonucleotide array comparative genomic hybridization analysis (B) [22]. (A) A complex duplication of the *PLP1* region detected by outward facing polymerase chain reaction. Panel (i) shows the *PLP1* region with the positions of the outward facing primers. The structure of the duplicated region is shown in (ii), with an enlargement of the complex junction region in (iii). Two or three bp of microhomology, shown by the letters A, C, G and T, were found at the breakpoint junctions (open arrows). (B) Deletion and duplications found in two patients with Pelizaeus-Merzbacher disease and their carrier mother [24], shown by comparative genomic hybridization. A ∼190-kb deletion is followed by a ∼9-kb segment with no copy-number change, and an interrupted ∼190-kb duplication was detected (i). Panel (ii) shows enlargement of the array revealing interruption of the ∼190-kb duplication. In each horizontal yellow box above, blue lines represent an average of the data points. Red data points indicate copy-number gains, green data points indicate losses, and black data points indicate no copy-number change. The *y*-axes show relative hybridization; genomic position is on the *x*-axis. Panel (iii) summarizes the structure based on comparative genomic hybridization where a green box shows the region deleted, red boxes show the regions duplicated, and black lines show regions of no change.

**Figure 3:**
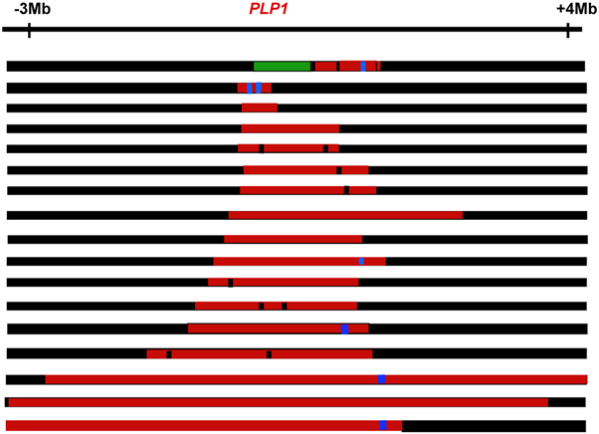
Complex genomic rearrangements at *PLP1* seen in patients with Pelizaeus-Merzbacher disease, illustrating long-range as well as short-range complexity. Duplications are shown in red, deletions in green, triplications in blue, and no copy number change in black. The figure is not drawn to scale. Approximate positions are given relative to *PLP1*.

Pelizaeus-Merzbacher disease (PMD; Online Mendelian Inheritance in Man (OMIM) accession code 312080; http://www.ncbi.nlm.nih.gov/omim/) is a recessive X-linked genomic disorder affecting the central nervous system that arises by nonrecurrent chromosomal changes. The changes involve duplication, triplication, or deletion of the *PLP1* gene. The clinical phenotype allows identification of individuals showing nonrecurrent chromosomal changes in the *PLP* region. In a study of the structural variation in the genomes of patients with PMD, Lee et al. [22] describe some aspects of the fine structure of newly arising CNVs with nonrecurrent endpoints and report three striking properties of their structure that help us to understand the origin of CNVs. First, the authors report that the novel junctions form at sites of microhomology, i.e., lengths of homology 2 to 5 nucleotides long that are too short to support homologous recombination. Such junctions have been reported previously in cases of nonrecurrent endpoints of deletions and duplications [19],[23],[24]. Second, they observed that the new structures are complex, showing duplication and deletion interspersed with nonduplicated or with triplicated lengths, and showing duplicated segments in either orientation. These characteristics were reported previously [25]–[31]. Third, although these events did not arise by NAHR, the novel junctions tend to occur in close proximity to LCRs [32]–[34]. Figures 2 and 3 illustrate examples of these complex non-recurrent events. Nonrecurrent rearrangements had previously been attributed to a mechanism of nonhomologous end-joining (NHEJ) [19],[20],[24],[33]. However, the characteristics of microhomology junctions and structural complexity in these new structures, as revealed by nucleotide sequencing and high-resolution array comparative genomic hybridization, led Lee et al. [22] to propose that the rearrangements arose through a replication-based mechanism termed FoSTeS (fork stalling and template switching), a mechanism proposed previously for amplification in *E. coli* [35]. Replication-based models have also been proposed to explain the origin of gross chromosomal rearrangements seen in a low proportion of patients with cystic fibrosis and hemophilia A. Analysis of deletions of the genes involved reveals complex structures similar to those described for *PLP1* [28],[29],[31].

## Genome Rearrangements in Cancer

The amount of structural variation in cancer cells is sometimes so extreme [36] that it is not possible to determine which changes occurred within the same event. However, it can be seen that duplications are often discontinuous, and junction regions include insertions of nearby, unlinked, and unknown sequences, and deletions and inversions [37], showing that rearrangement events in cancer cells are complex. Many studies report microhomology at junctions of a large proportion of the structural variation (e. g., [37]–[39]). Studies of translocation endpoints in leukemia and other cancers find that many junctions have microhomology and are associated with insertions and deletions of various lengths [40]–[42]. These observations are compatible with at least some of the genomic instability seen in tumor formation and progression having stemmed from the same underlying mechanism as the formation of nonrecurrent duplications in genomic disorders.

## Involvement of Replication in Chromosomal Structural Change

In the Lac assay system in *E. coli* [43], amplification of the *lac* operon to 20–100 copies occurs in response to the stress of starvation [44],[45]. The novel junctions of the amplified segments (amplicons) show that endpoints occurred at sites of microhomology of 2–15 bp [35],[46]. Some of the amplicons are complex, containing both direct and inverted repeats. Many others cannot be identified by outward-facing polymerase chain reaction (an observation also encountered frequently for *PLP1* duplication junction analysis [22]), which would reveal the junctions of simple tandem repeats, and so are presumed to be complex, rather than simple tandem repeats [35],[46],[47]. By these criteria, about 25% of amplicons are complex. Thus, with respect to microhomology and complexity, the chromosomal structural changes in this system resemble those found in nonrecurrent events in human genomic disorders.

Homologous recombination requires RecA protein (Rad51 in eukaryotes) (reviewed in [48]). Microhomology-mediated deletion formation in *E. coli* (less than 25 nucleotides of homology) has long been known to be RecA-independent [49]–[52]. RecA-independent short homology-mediated deletions (25–50 nucleotides) have previously been attributed to template switching within a replication fork during DNA replication (reviewed in [53]). The evidence for this is, first, that mutations in genes encoding replication functions affect the formation of these events; second, that mutations affecting post-replicational mismatch repair affect them, placing the event very near to the replication fork; third, that mutation of 3′ exonucleases has an effect that is consistent with the ends being used to prime DNA synthesis; and fourth, that it is very difficult to obtain mutations affecting the process by transposon mutagenesis, suggesting essential functions.

In the *E. coli* Lac system, study of genetic requirements of stress-induced amplification has revealed some details of the mechanism. First, the events involve 3′ DNA ends. This is seen by an increase in amplification when a 3′ exonuclease gene (*xonA*) is deleted, and a decrease when the 3′ exonuclease is over-expressed. Similar manipulation of 5′-exonuclease has no effect [35]. This suggests that amplification results from free 3′ ends in the cell most of which are normally removed by exonuclease. As above, the involvement of 3′ ends but not 5′ ends is consistent with priming of DNA synthesis.

Second, lagging-strand processing at replication forks is implicated by a requirement for the 5′ exonuclease domain of DNA polymerase I (Pol I) [35],[45]. Pol I is involved in lagging-strand replication, base excision repair, and nucleotide excision repair, but these excision repair processes are not involved in amplification [35], so lagging strands at replication forks are implicated in amplification.

Third, there is a requirement for the proteins of double-strand break (DSB) repair by homologous recombination [35] (the RecBC system, reviewed in [48]). That this is actually a requirement for DSB repair (not just the proteins) is shown by the discovery that in vivo double-strand cleavage of DNA near *lac* enhances amplification rates [54].

Taken together, these observations suggest a model for amplification in the Lac system in *E. coli* in which replication is restarted at sites of repair of DNA double-strand ends [35]. The hypothesis proposed was that template switching occurs during replication restart at stalled replication forks. Because the distances involved exceed the lengths that are expected to be exposed as single-stranded at a single replication fork, it was proposed that the switches occurred between different replication forks [35].

The idea that chromosomal structural changes originate from DNA replication has received support from a study of microhomology-mediated SD formation in yeast [55]. These authors support the idea that the mechanism of SD formation involves replication by showing that its frequency is enhanced by treatment with camptothecin and is dependent on Pol32, a component of Polδ (discussed below). Camptothecin is a topoisomerase I inhibitor that leaves nicks in DNA. These nicks are believed to become collapsed forks when a replication fork reaches them. Thus, increasing the frequency of fork collapse increases the frequency of duplication formation. These authors also report that situations that lead to fork stalling rather than collapse have little effect on the frequency of duplication formation [55]. Thus, it appears that the substrate for duplication is a single double-strand end at a collapsed replication fork.

This long-distance template-switch model was also used by Lee et al. [22] to explain the observations of nonrecurrent chromosomal changes seen in Pelizaeus-Merzbacher disease discussed above and the juxtaposition of multiple genomic sequences normally separated by large genomic distances [22],[56]. Experiments on the integration of nonhomologous DNA into mammalian cells revealed microhomology junctions and insertion of sequence from other parts of the genome at the junctions. These observations were interpreted in terms of a similar model of repeated copying and switching to another template [57].

## Break-Induced Replication

**Figure 4:**
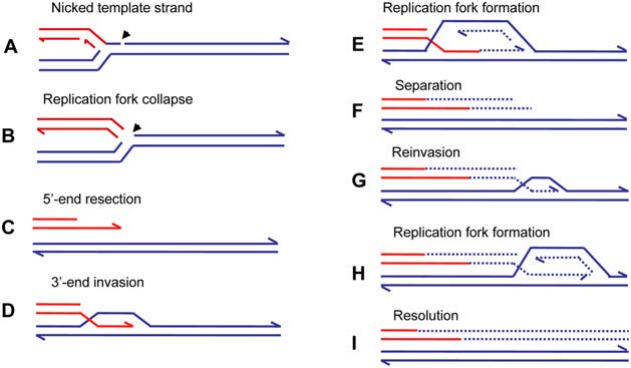
Repair of a collapsed replication fork by BIR. When a replication fork encounters a nick in a template strand (A) (arrowhead), one arm of the fork breaks off (red), producing a collapsed fork (B). At the single double-strand end, the 5′ strand is resected, giving a 3′ overhang (C). The 3′ single-strand end invades the sister molecule (blue), forming a D-loop (D), which subsequently becomes a replication fork with both leading and lagging strand replication (E). There is a Holliday junction at the site of the D-loop. Migration of the Holliday junction, or some other helicase activity, separates the extended double-strand end from its templates (F). The separated end is again processed to give a 3′ single-strand end, which again invades the sister, and forms a replication fork (G). Eventually the replication fork becomes fully processive, and continues replication to the chromosome end (H and I). This process is shown here with the Holliday junction following the fork so that newly formed strands are segregated together (conservative segregation) (H). Each line represents a DNA nucleotide chain (strand). Polarity is indicated by half arrows on 3′ end. New DNA synthesis is shown by dashed lines. The publications on which this model is based are cited in the text.

A more specific model for restarting replication at collapsed (broken) replication forks, BIR [58], has been developed for yeast, and a similar mechanism was proposed to explain telomere maintenance in yeast and human cell lines that have lost telomerase activity (reviewed in [59]). Recent evidence [60],[61] suggests that the BIR mechanism can be modified to explain the complexity of chromosomal structural changes described above for human and *E. coli*. Figure 4 illustrates the mechanism of BIR. When the replicative helicase encounters a nick on the template strand (Figure 4A), one arm of a replication fork breaks off (Figure 4B). There is no second end to be involved in the mechanisms of DSB repair that are available at a DSB consisting of two double-strand ends: homologous recombination or nonhomologous end-joining. The 5′ end of the broken arm is resected by an exonuclease to leave a 3′ overhang (Figure 4C). This 3′ tail invades a homologous sequence, normally the sister chromatid from which it came. This invasion is mediated by RecA/Rad51 protein (Figure 4D). The 3′ end primes DNA synthesis and establishes a replication fork consisting of both leading and lagging strand synthesis [61] (Figure 4E). This replication is of low processivity, and the extended arm is separated from the sister chromatid (Figure 4E). Such separation might be achieved by migration of the Holliday junction shown in Figure 4D and 4E. The 3′ end reinvades and the process is repeated (Figure 4G and 4H). After a few cycles of invasion, extension, and separation, the replication fork becomes more processive, and replication continues to the end of the chromosome arm or to the end of the replicon. The change from low processivity to highly processive replication can be attributed to a switch in the DNA polymerases involved [61]. Initial extension from a double-strand end was shown to require the primase complex and Polδ, notably the nonessential Pol32 subunit, whereas the more processive Polε was required for the 30-kb extension to the telomere. Figure 4I shows the completed pair of chromatids with the new material segregating conservatively as suggested for *E. coli* [62]. This would result if the Holliday junction followed the replication fork. Another possibility is that the Holliday junction is resolved so that there will be semi-conservative segregation of old and new DNA strands [60], (reviewed in [63]). Evidence for conservative segregation of new DNA strands in BIR, suggesting that the Holliday junction was not resolved, was reported for *E. coli* [62].

The repeated extension and separation have been interpreted as repeated attempts to find the other side of a break consisting of two double-strand ends. When, eventually, none is found because this is a collapsed fork rather than a two ended DSB, the remainder of the chromosome is replaced by replication [60],[63]. The pattern of repeated rounds of template switching followed by a long length of replication is supported by observations of BIR in yeast. BIR can be induced experimentally by transforming a chromosomal fragment into a yeast cell [64]. Using such a system, Smith et al. [60] placed a chromosomal fragment with a centromere and one telomere-forming sequence into a diploid yeast cell. The fragment had homology to both homologues of chromosome III. These homologues were differentially marked. Selection for a marker on the fragment selected for cells in which the fragment had acquired a second telomere. These authors found that most fragments had completed the replication of 50 kb to the end of the chromosome to which the fragment had homology. The striking result was that many of the chromosomes recovered had switched from one homologue to the other. In some cases, more than one switch was seen. The switches were confined to the first 10 kb, after which a single homologue was copied. In a few percent of cases, the switch was to a different chromosome at sites of repeated homology consisting of the long terminal repeat of a retrotransposon. Thus, BIR was demonstrated to produce complexity of the sorts reported above for *E. coli* amplification and for nonrecurrent end-points in human genomic disorders.

BIR has been suggested as the mechanism that underlies SD and other structural changes in yeast, e.g., [55],[65],[66], and human, e.g., [31],[67]. As discussed below, BIR is strongly RecA/Rad51-dependent and homology-dependent, and so cannot account for the observations of microhomology associated with complex rearrangements without substantial change.

## Microhomology-Mediated BIR (MMBIR)

BIR, as described above, is usually an accurate process, because the repeated invasions are RecA/Rad51-mediated and involve long lengths of homology between DNA sequences. Invasion catalyzed by RecA/Rad51 requires extensive homology of about 50 bp in *E. coli* [68] and more in eukaryotes [69],[70]. This does not fit with the microhomology junctions described above. We therefore suggest that in these systems, replication forks are reestablished in a RecA/Rad51-independent manner. Rad51-independent BIR occurs in yeast at a much lower efficiency than the Rad51-dependent BIR [71],[72], though its frequency is very much enhanced, at the expense of fidelity, by the presence of unusual structures such as an inverted repeat [71]. However, telomere recombination in the absence of telomerase is proficient in the absence of Rad51 and is mediated by very short homologies [73],[74] (reviewed in [59]). The fact that telomere recombination occurs by BIR is supported by the finding that it requires the same set of enzymes as BIR that is initiated in the middle of a chromosome [61]. Absence or shortage of RecA/Rad51 might arise because the cells are stressed, as described below. That microhomology-mediated SD formation occurs in yeast by a BIR mechanism is supported by the finding that, like homology-mediated BIR [61], it requires Pol32 [55].

In mammalian cells, there is a surprisingly efficient microhomology-mediated DSB repair pathway. Most, if not all, experimental research on microhomology-mediated DSB repair has been performed with nuclease-induced breaks. This recently described pathway was characterized in recombination events induced by I-SceI or RAG1/RAG2 nucleases in cells deficient in classical NHEJ and in cancer cells [75],[76]. Nucleases generate two-ended breaks at random with respect to ongoing replication forks. However, BIR acts under circumstances when DSB repair, including NHEJ, is not an option, because after replication fork breakage, there is only a single end with no second end to which the one end can be annealed or ligated. Spontaneous damage to DNA occurs predominantly during replication [77]–[79], so that mechanisms that repair single DNA ends are more appropriately invoked for spontaneous damage than are mechanisms that act on two-ended DSBs. We suggest that a novel pathway, microhomology-mediated BIR (MMBIR), is used to repair single double-strand ends when stretches of single-stranded DNA are available and share microhomology with the 3′ single-strand end from the collapsed fork.

Single-stranded DNA might be expected to occur in replication forks, from stalled transcription complexes, at excision repair tracts, or at secondary structures in DNA such as cruciforms or hairpins caused by complex genomic architecture, and possibly in other situations such as in promoter regions and replication origins. The dimensions of most of the template switches discussed here (tens to hundreds of kb distant, i.e., the length of a duplication or deletion) preclude mechanisms of replication slippage within a single replication fork. An ability of any single-stranded DNA region that shares microhomology with the single-stranded 3′ end to take part in the events would explain why MMBIR is inexact and liable to lead to chromosomal structural changes. Very short homology should not be a barrier to replication fork restart because polymerase eta, used in DSB repair in vertebrates [80],[81], is efficient in initiating new DNA synthesis from mismatched primers, and even primers as short as 2–3 bp [82].

**Figure 5:**
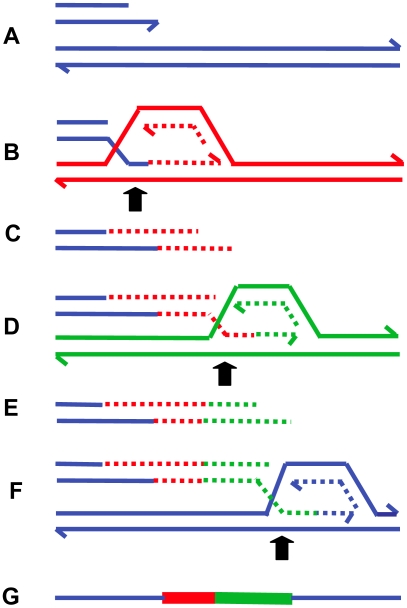
MMBIR. The figure shows successive switches to different genomic positions (distinguished by color) forming microhomology junctions (arrows). For clarity, the nature of the single-stranded regions of annealing is not defined (see text). (A) shows the broken arm of a collapsed replication fork, which forms a new low-processivity fork as shown at (B). The extended end dissociates repeatedly ((C and E) shown with 5′-ends resected) and reforms the fork on different templates (D and F). In (F), the switch returns to the original sister chromatid (blue), forming a processive replication fork that completes replication. (G) shows the final product containing sequence from different genomic regions. Each line represents a DNA nucleotide chain (strand). Polarity is indicated by half arrows on 3′ end. Whether the return to the sister chromatid occurs in front of or behind the position of the original collapse determines whether there is a deletion or duplication (see Table 2).

The presence of inverted repeats could generate hairpin loops that expose single-stranded sequence [22],[32]. In addition, hairpin structures might increase the likelihood of replication fork stalling, which might then initiate BIR. Such major roles for secondary DNA structures in the generation of chromosomal structural changes offers an explanation for the clustering of structural changes, producing complex chromosomal regions such as that illustrated in Figure 1. The model of MMBIR is presented in Figure 5.

The clear distinction between NHEJ and BIR mediated by microhomology is that, in the second instance, microhomology junctions are followed by shorter or longer stretches of DNA sequence derived from elsewhere. Ten to 20% of nonhomologous junctions in mammalian cells have sequence inserted at the junction [83]. Some events that had previously been interpreted as occurring by an NHEJ mechanism might have occurred by MMBIR with a single template switch. In addition, events that appeared to be simple end-joining events might have had complexity that was not revealed by the techniques in use.

## Control of BIR and MMBIR

A major question remains—why do cells use microhomology- and not homology-driven repair? The likely answer is that Rad51 is not available or is in short supply. This might be caused by stress responses. Evidence supporting this comes from cancer research. Hypoxia in the tumor microenvironment is correlated with genetic instability [84],[85] (reviewed in [86]). It has been shown that hypoxia leads to repression of *RAD51* and *BRCA1* [87],[88] and to reduced homologous recombination [87],[89] (reviewed in [89],[90]). This has been interpreted as a switch from high-fidelity homologous recombination to lower fidelity NHEJ caused by stress [87],[88]. At collapsed replication forks, where NHEJ is not possible, we suggest that down-regulation of *RAD51* prevents BIR from following the Rad51- homology-dependent BIR route but still allows a Rad51-independent BIR route that requires very much less homology, as observed in telomere recombination in budding yeast [73],[74]. If Rad51 is down-regulated but not absent, a condition might prevail in which some homologous invasion is allowed, but not enough to prevent some illegitimate events occurring, as was witnessed in *Drosophila* with reduced gene dosage of Rad51 [91]. We do not know whether the error-prone nature of this repair is aided by down-regulation of mismatch repair, which has also been reported for stressed cancer cells [92],[93]. There might be other changes in gene expression under stress that promote genomic instability (e.g., [94]).

A similar switch from high fidelity to low-fidelity DSB repair is seen in *E. coli* in response to the stress of starvation [54]. Similarly the microhomology-mediated amplification seen in the Lac system in *E. coli* discussed above is induced by stress, as evidenced by the observation that the event occurred after the beginning of starvation [44], and by the finding that adaptive amplification in this system requires the starvation and general stress response transcriptional activator RpoS [95].

The mechanism of MMBIR, as described above, features annealing of single-stranded DNA with minimal homology. Hence the enzyme responsible for this has a central role in the proposed mechanism. We suggest that annealing is catalyzed by Rad52. Rad52 is essential for the single-strand annealing reaction that deletes sequence between direct repeats [96], and it anneals single strands in vitro [97]. Chromosomal rearrangements in yeast that have microhomology at the junctions have been seen to occur in the absence of Rad51, but they require Rad52 [42],[66],[98]. In one of these cases, frequent switches were associated with microhomology junctions in a Rad51-independent, Rad52-dependent process that produced translocations and inversions at sites of highly diverged genes [66]. These authors proposed that these events occurred by template switching during BIR [66]. In vitro, Rad51 inhibits the single-strand annealing activity of Rad52 [99], suggesting that the absence of Rad51 might exercise tight control of the switch from strand invasion to annealing of single strands. However, the formation of microhomology-mediated Rad51-independent SDs in yeast was found to be Rad52-independent [55]. Rad52 is also not required for microhomology-mediated end-joining [100]. These observations show that microhomology junction formation can be mediated by a different protein in yeast, as well as by Rad52.

In summary, we are suggesting that, because stress induces a reduction in the amount of Rad51 available, while leaving Rad52 unchanged, the amount of homologous interaction that is used for repair is reduced, leaving annealing of single DNA strands as the main mechanism available for the repair of collapsed replication forks. Thus, classical BIR will be reduced, and MMBIR will be substituted.

## Long-Range Discontinuities in Duplications

The idea that there is a cell-wide physiological condition that favors nonhomologous interactions has further implications. If a condition prevails that allows one such event, it is possible that further nonhomologous events will occur in the same cell. The possibility of multiple rounds of events was suggested for a yeast system to correct for an inversion that would produce a dicentric chromosome [66]. We also note that, in human duplications, there are discontinuities (short regions that are not duplicated) and triplicated regions within duplications on a scale of hundreds of kb or Mb apart (Figures 2 and 3). These long-distance interruptions are not readily explained by template switching during the early stages of a single BIR event, where switching occurs after one template is copied for hundreds of bp to a few kb (Figure 2 and [60]), but rather suggest that more than one BIR event occurred along the same chromosome. MMBIR requires, in addition to a cell-wide stress response, a specific DNA structure: a single double-strand end. To explain why single double-strand ends should occur serially along the same chromosome, we propose that the Holliday junction formed during BIR follows the replication fork, as we have suggested above as the mechanism of separation of the extended broken end. If the replication fork formed by BIR stalls for any reason, the Holliday junction might then process through the fork, separating the newly synthesized DNA from its template, and so generating a collapsed fork anew (as in Figure 4E and 4F) and leading to the long range discontinuities seen in duplicated segments, as illustrated in Figure 3.

## Chromosome Structural Consequences of MMBIR

The ways in which MMBIR would lead to the various chromosomal structural changes are summarized in Table 2. Translocations would be formed by a switch to a different chromosome. Duplication would occur when the switch was to either the sister or the homologue behind the position at which the fork collapsed (with respect to the direction of movement of the fork). Deletion happens when there is a switch to a position ahead of the fork collapse. A switch to a sequence that has already been duplicated, behind the end of the duplicated sequence, would produce a triplication. Switching to the same molecule behind the position of fork collapse has the potential to initiate rolling-circle replication and consequent amplification. Switching to either the sister molecule or the homologue in inverted orientation would give an inverted chromosomal segment. If long-distance replication follows, this might form a dicentric chromosome, so that this would have to be followed by a second inversion to allow a cell to be viable. This need for a second switch has led to the idea that there might be more than one round of switching events involved in the formation of some structural changes [66] as discussed above. Alternatively, a second inverted template switch within a single series of switches would restore a viable chromosomal structure.

**Table 2:** Chromosomal Consequences of Template Switches during MMBIR.

When Switch Is to:	Consequence:
Sister or homologue behind position of fork breakage	Duplication
Sister or homologue ahead of position of fork breakage	Deletion
Sister or homologue in wrong orientation	Inversion
Nonhomologous sequence	Translocation
Sequence already duplicated	Triplication
On same molecule behind the break	Rolling circle

## Implications of the Model

We suggest that the replicative mechanism described here contributes to genomic disorders that show nonrecurrent endpoints, contributes to much of the chromosomal structural instability that occurs somatically in cancer formation and tumor progression and also to the origin of the genomic constitutional structural complexity that underlies NAHR genomic disorders, and is a driving force in evolution. We offer evidence from diverse organisms that such a mechanism exists, and suggest that the model offers directions for future research that will further elucidate the molecular details.

The mechanism of MMBIR affects human biology at many levels. First, at the cellular level, the mechanism might apply to the events underlying much cancer formation and progression. Second, at the organismal level, we propose that MMBIR acting in the germline will give rise to CNV, and the accompanying genomic disorders and chromosomal syndromes. At the same time MMBIR could create LCRs that provides the homology required for NAHR, leading to genomic disorders in future generations. Third, at the species level, we suggest that complex genomic regions generate secondary structures that increase the likelihood of MMBIR, so that complex architecture becomes more complex on an evolutionary timescale, as has been documented for primate evolution [13],[16]. We suggest that MMBIR might underlie genomic rearrangements and CNV associated with the emergence of primate-specific traits [10],[13],[101]. Furthermore, MMBIR provides material on which natural selection and evolution operate: variation in copy number might change the expression levels of included genes and also provide redundant copies of genes that could then be mutated and changed to encode new functions [102]–[104]. Further, the formation of nonhomologous junctions might shuffle exons of different genes to attain new functions (F. Zhang and J. Lupski, unpublished observations). Indeed, these regions of complex genomic architecture have been referred to as gene nurseries, i.e., regions in which new genes are formed [13],[14].

The MMBIR model predicts that complex genomic rearrangements will often be accompanied by extensive loss of heterozygosity and, in some cases, by loss of imprinting, because the chromosome that is copied might be either the sister or the homologue. Such loss of heterozygosity could lead to regional uniparental disomy [105] as a potential novel mechanism for disease. We also predict that the events described here will be seen in model systems under conditions where the cells are stressed, and study of DNA repair activities in stressed cells might be a fertile field for investigation.
